# Spontaneous brain activity in healthy aging: An overview through fluctuations and regional homogeneity

**DOI:** 10.3389/fnagi.2022.1002811

**Published:** 2023-01-12

**Authors:** Marc Montalà-Flaquer, Cristina Cañete-Massé, Lídia Vaqué-Alcázar, David Bartrés-Faz, Maribel Peró-Cebollero, Joan Guàrdia-Olmos

**Affiliations:** ^1^Department of Social Psychology and Quantitative Psychology, Faculty of Psychology, Universitat de Barcelona, Barcelona, Spain; ^2^UB Institute of Complex Systems, Universitat de Barcelona, Barcelona, Spain; ^3^Institute of Neurosciences, Universitat de Barcelona, Barcelona, Spain; ^4^Department of Medicine, Faculty of Medicine and Health Sciences, Universitat de Barcelona, Barcelona, Spain; ^5^Institut d’Investigacions Biomèdiques August Pi i Sunyer (IDIBAPS), Barcelona, Spain

**Keywords:** spontaneous brain activity, resting-state functional magnetic resonance imaging, fractional amplitude of low-frequency fluctuation, regional homogeneity, healthy aging

## Abstract

**Introduction:**

This study aims to explore whole-brain resting-state spontaneous brain activity using fractional amplitude of low-frequency fluctuation (fALFF) and regional homogeneity (ReHo) strategies to find differences among age groups within a population ranging from middle age to older adults.

**Methods:**

The sample comprised 112 healthy persons (*M* = 68.80, SD = 7.99) aged 48–89 who were split into six age groups (< 60, 60–64, 65–69, 70–74, 75–79, and ≥ 80). Fractional amplitude of low-frequency fluctuation and ReHo analyses were performed and were compared among the six age groups, and the significant results commonly found across groups were correlated with the gray matter volume of the areas and the age variable.

**Results:**

Increased activity was found using fALFF in the superior temporal gyrus and inferior frontal gyrus when comparing the first group and the fifth. Regarding ReHo analysis, Group 6 showed increased ReHo in the temporal lobe (hippocampus), right and left precuneus, right caudate, and right and left thalamus depending on the age group. Moreover, significant correlations between age and fALFF and ReHo clusters, as well as with their gray matter volume were found, meaning that the higher the age, the higher the regional synchronization, the lower the fALFF activation, and the lower gray matter of the right thalamus.

**Conclusion:**

Both techniques have been shown to be valuable and usable tools for disentangling brain changes in activation in a very low interval of years in healthy aging.

## 1. Introduction

Life expectancy in the general population has significantly increased in the last few years thanks to an improvement in medicine and social services, among others (Sanchez-Morate et al., 2020). The percentage of older people is growing at a rate of 3% per year (United Nations, Department of Economic and Social Affairs, Population Division, 2017). Thus, there is a need to understand the underlying processes of healthy aging.

Healthy aging is the process of developing and maintaining functional ability that enables well-being in older age (World Health Organization, 2016). However, aging is also associated with deficits in areas such as executive function or in sensory functioning (Rosano et al., 2005), among others (Park and Reuter-Lorenz, 2009). Recent longitudinal studies have shown that these deficits linked with healthy aging predict functional disability, future falls, or the onset of dementia among nondemented older adults (Johnson et al., 2009; Ewers et al., 2014; Veldsman et al., 2020). Therefore, understanding age-related changes in brain functioning could provide insights into the mechanisms underlying age-related functional declines and contribute to the actual interventions for cognitive improvements in the elderly population.

In the last few years, interest in neuroimaging studies has grown substantially. Several methods in this field, such as structural and functional connectivity (FC), have shown promising results in healthy aging (Oschmann et al., 2020; Yamashita et al., 2021; Abellaneda-Pérez et al., 2022). It is important to note that even for healthy individuals, the aging process implies changes in functional and structural connectivity (Farras-Permanyer et al., 2019; Oschwald et al., 2020; Vaqué-Alcázar et al., 2020). Regarding functional findings, aging has been associated with a decrease in FC in the default mode network (DMN) (Mowinckel et al., 2012), among other abnormalities (Chen, 2019; West et al., 2019). Aging has also been associated with decreased gray matter volume in frontal and parietal lobes (Hu et al., 2014), and these regions have also been highlighted as the most vulnerable to aging (Marchitelli et al., 2018). However, Yang et al. (2019) performing a multimodal neuroimaging analysis suggested that effect of age difference is not limited to only the frontal lobe region but in more widespread range, involving nonfrontal regions such as parietal, occipital, cuneus, and parahippocampal, among others.

The spontaneous blood-oxygen-level-dependent (BOLD) signal provides an indirect measure of the brain’s hemodynamics (Gorges et al., 2014), and its FC can be extracted (Ystad et al., 2011). However, FC usually depicts the relationship between two or more areas but does not provide detailed information on which exact voxels are abnormal within networks. Moreover, as the brain ages, several brain regions and connectivity networks could be altered in terms of dynamics and location, decreasing the accuracy of regions of interest (ROIs) and seed-based analyses (Lee and Hsieh, 2017). In contrast, regional spontaneous brain activity analysis may provide this helpful information (Zou et al., 2008) and thus could help disentangle differences in regional activities (Zang et al., 2015). The functional coordination between brain areas can be assessed through the BOLD signal by analyzing the amplitude of low-frequency fluctuations (ALFF) and its regional homogeneity (ReHo).

Amplitude of low-frequency fluctuations and ReHo are data-driven analyses of the brain signal that reveal different regional characteristics of resting-state functional magnetic resonance imaging (rs-fMRI) data; hence, they require no hypotheses or a priori selection of brain (ROI) (Lee and Hsieh, 2017). Amplitude of low-frequency fluctuation measures the correlation of local amplitude of spontaneous low-frequency fluctuations in the BOLD time series (frequency-domain analysis) (An et al., 2013), whereas ReHo computes Kendall’s coefficient of concordance (KCC) to assess the temporal synchronization (time-domain analysis) given a cluster of neighboring voxels (Zang et al., 2004). As ALFF appears to be sensitive to physiological noise, Zou et al. (2008) proposed the fractional amplitude of low-frequency fluctuations (fALFF), a low-pass filter of ALFF that enhances its sensitivity and specificity.

Both approaches may be complementary (Lee and Hsieh, 2017). They have been recently used in many psychiatric diseases (Lai et al., 2020; Gao et al., 2021; Wang et al., 2022), dementias (Liu et al., 2014; Yue et al., 2020), and healthy populations (Hu et al., 2014; Deng et al., 2022). Cha et al. (2015) performed an interesting meta-analysis studying functional abnormalities in amnestic mild cognitive impairment and Alzheimer’s disease (AD) patients using ReHo and fALFF, among other techniques, and they found decreased functional characteristics with all approaches. The results showed that the functional characteristics in the left parahippocampal gyrus were decreased in AD patients compared with healthy subjects. Hu et al. (2014) studied fALFF during a stop signal task in a healthy aging sample and found a negative correlation with age in some areas of the frontal and prefrontal regions, among others, indicating that spontaneous neural activities in these areas decrease with age while performing a task. Hsu et al. (2020) used a graph theoretic perspective similar to fALFF and ReHo to examine the relationship between properties of topological organization in functional brain networks and motor inhibition and found the implication of frontoparietal regions among others.

Farras-Permanyer et al. (2019), in a study with the same population as the one used in this study, found a progressive decrease in FC between six groups of healthy aged individuals and was particularly pronounced concerning the group aged between 75 and 79 years old. Furthermore, the oldest group showed a slight increase in FC and was interpreted as a compensatory mechanism in brain functioning.

Although age is markedly related to changes in functional and anatomical connectivity when analyzing the whole-brain BOLD signal some results from different investigations are still inconsistent. Additional studies that expand upon data-driven analyses could provide supplementary information about the different regional characteristics of the brain in healthy older adults. Moreover, the spontaneous activity of age-related brain networks can be an effective indicator of individual differences and age-group differences in elderly people (Lee and Hsieh, 2017). fALFF and ReHo show remarkably high temporal stability and long-term test–retest reliability (Zuo and Xing, 2014). Consequently, both techniques have been suggested to be potential biomarkers (Küblböck et al., 2014; Zuo and Xing, 2014).

The present paper aims to study the whole-brain resting state using fALFF and ReHo strategies to find differences in spontaneous brain activity among healthy participants of different age groups from middle to advanced age. Despite the incongruencies between the studies, we predict differences in fALFF and ReHo in the frontal lobe and in the DMN. Farras-Permanyer et al. (2019) already demonstrated differences between these age groups in FC whereas spontaneous brain activity remained unexplored. Therefore, we also hypothesize to find differences between these age groups in fALFF and ReHo in the same line as Farras-Permanyer et al. (2019).

## 2. Materials and methods

### 2.1. Participants

The original data used in this study are the same as those used in Farras-Permanyer et al. (2019) comprised by rs-fMRI sequences of 121 healthy individuals merged from three different studies conducted at the Department of Medicine, School of Medicine and Health Sciences, University of Barcelona. However, two subjects were discarded because the T1-weighted acquisition was noisy, four subjects were excluded due to excessive movement during the registration (Jenkinson et al., 2002) and four subjects were excluded due to incomplete recordings, leaving a total sample of 112 participants. The three protocols were approved by the ethics committee from the Comissió de Bioètica of the Universitat de Barcelona (Approval No. PSI2012-38257) and the ethics committee from Barcelona’s Hospital Clínic (Approval No. 2009-5306 and Approval No. 2011-6604).

The exclusion criteria included illiteracy or an inability to understand the protocol or undergo neuropsychological tests mentioned in the next section, prior cerebrovascular accident, any relevant psychiatric illness, advanced cognitive deterioration, dementia, or other neurodegenerative diseases (e.g., Parkinson’s disease), any chronic illness expected to shorten survival (grave diseases such as heart failure, chronic liver disease, kidney failure, blood disease or cancer) and any MRI-related incompatibility (the presence of metallic objects within the body, pacemaker or claustrophobia).

The final participant sample comprised 112 healthy individuals aged 48–89 years (68.80 ± 7.99) years (50% females). The participants were split into 6 age groups (< 60, 60–64, 65–69, 70–74, 75–79, and ≥ 80) with the following group sizes: (*n*_1_ = 12; *n*_2_ = 21; *n*_3_ = 30; *n*_4_ = 21; *n*_5_ = 18; and *n*_6_ = 10). The age intervals were chosen to detect slight differences in relatively short aging intervals. This categorization had been used in previous studies and is coherent with the suggestion made by Sala-Llonch et al. (2015). Only two participants were younger than 55 years in the first group, and only four participants were older than 85 years.

### 2.2. Instruments

The three protocols contained a neuropsychological assessment of major cognitive domains, including the vocabulary scale in the Wechsler Adult Intelligence Scale (WAIS) (Lezak et al., 2004), the mini-mental state examination (MMSE) (Folstein et al., 1975; Tombaugh and McIntyre, 1992), the National Adult Reading Test (NART; Nelson and Willison, 1991) and the Boston Naming Test (BNT) (Kaplan et al., 2001).

However, specifically, the participants of the first and third protocols were also assessed with the Rey Auditory Verbal Learning Test (n = 80; Rey, 1964), and the participants of the second protocol were also assessed with the Grober and Buschke Test (*n* = 32; Grober and Buschke, 1987).

### 2.3. Magnetic resonance imaging acquisition and preprocessing

The three protocols used a Siemens Magnetom Trio Tim syngo 3-T system scanner at the Unitat d’Imatge per Ressonància Magnètica IDIBAPS (Hospital Clínic), Barcelona. First, a high-resolution T1-weighted structural image was obtained with a magnetization-prepared rapid acquisition gradient echo (MPRAGE) three-dimensional protocol with repetition time (TR) = 2,300 ms, echo time (TE) = 2.98 ms, 240 slices, slice thickness = 1 mm, and field of view (FOV) = 256 mm. For the resting-state acquisition, participants were instructed to lie down with their eyes closed and not fall asleep. Notably, the BOLD signal acquisition was slightly different for each protocol:

- Protocol 1: *n* = 32 participants, TR = 2,000 ms, TE = 16 ms, slice thickness = 3 mm, interslice gap = 25%, FOV = 220 mm, total: 5 min. Ethics committee of the Comisión de Bioética de la Universidad de Barcelona, approval number: PSI2012-38257.
- Protocol 2: *n* = 57 participants, TR = 2,000 ms, TE = 16 ms, slice thickness = 3 mm, interslice gap = 25%, FOV = 220 mm, total: 10 min. Ethics committee of the Barcelona’s Hospital Clínic, approval number: 2009-5306.
- Protocol 3: *n* = 23 participants, TR = 2,000 ms, TE = 19 ms, slice thickness = 3 mm, interslice gap = 25%, FOV = 220 mm, total: 5 min. Ethics committee of the Barcelona’s Hospital Clínic, approval number: 2011-6604.

While Protocols 1 and 3 recorded 150 dynamic points, Protocol 2 recorded a total of 300 dynamics. This difference between protocols can complicate the statistical processing of the data, so the temporal registries of Protocol 2 were truncated, and only the first 150 dynamical points were used. Additionally, a difference in the echo time (TE) on Protocol 3 was reported, but it was so slight that no further effect was seen on the sample data between protocols.

The structural T2 images of every participant were revised to identify any possible abnormality before including it in the statistical analysis. No structural abnormalities or alterations were found in any participant.

### 2.4. Voxel-based morphometry

The T1w-structural images were automatically processed with DPABI (Yan et al., 2016). The images were reoriented and individually checked for quality control. Afterwards, reoriented T1 images were segmented into gray matter (GM), white matter (WM) and cerebrospinal fluid (CSF; Ashburner and Friston, 2005). Finally, the DPABI module uses the Diffeomorphic Anatomical Registration Through Exponentiated Lie algebra (DARTEL) tool (Ashburner, 2007) to compute transformations from individual native space to MNI space. Finally, gray matter segmentations where resliced and smoothed to match the parameters with the functional images. Additionally, total gray matter volumes and parcellation volumes were calculated using SPM12^1^ and SPM12 based scripts (Maldjian et al., 2003, 2004).

### 2.5. Data preprocessing

Image preprocessing was performed using the Data Processing Assistant for Resting-State fMRI (DPARSF; Yan and Zang, 2010).^2^ Essentially, the pipeline is based on MATLAB, SPM12 and DPABI.

Primarily, the first ten functional images were discarded to avoid possible effects from participants adapting to the scanner and to let the magnetization equilibrate properly. Then, the remaining functional images were corrected for slice time by means of their timing acquisition, and head motion was assessed. Nuisance signals were regressed out considering white matter and cerebrospinal fluid signals, linear trends and, finally, signals associated with the 24 Friston head-motion parameters (Friston et al., 1996). The derived functional images were coregistered with their corresponding structural images, which were segmented and normalized to MNI space using DARTEL tool. The functional images were also normalized to MNI space with warped parameters and resampled to 3 mm cubic voxels. With regard to the ReHo analysis, the normalized functional images were then bandpass filtered (0.01–0.1 Hz). To assess excessive movement from the functional recording, participants exceeding the group mean plus two standard deviations (Yan et al., 2013) were excluded from the study. The mean movement group value was estimated with Jenkinson’s framewise displacement (FD; Jenkinson et al., 2002), and the mean FD is shown in Table 1 for each group. As mentioned in the participants section, 10 participants were discarded, and the final sample was 112 healthy people. Therefore, further statistical analyses were performed with the covariate of mean Jenkinson’s FD for every subject.

**Table 1:** Description of movement and neuropsychological measures between age groups.

Age groups ( $\bar{x}\pm \mathrm{SD})$ (years)	Size	FD Jenkinson ( $\bar{x}\pm \mathrm{SD})$ (mm)	BNT ( $\bar{x}\pm \mathrm{SD})$	NART ( $\bar{x}\pm \mathrm{SD})$	WAIS-Voc ( $\bar{x}\pm \mathrm{SD})$	MMSE ( $\bar{x}\pm \mathrm{SD})$
< 60 (54.67 ± 3.91)	12	0.24 ± 0.12	55.50 ± 5.22	25.17 ± 3.81	40.22 ± 12.70	*29.33 ± 0.89*
60–64 (62.29 ± 1.35)	21	0.22 ± 0.12	54.81 ± 2.94	24.86 ± 3.45	47.62 ± 16.11	28.76 ± 1.09
65–69 (67.13 ± 1.28)	30	0.23 ± 0.14	57.07 ± 8.77	28.93 ± 3.58	46.36 ± 10.75	29.31 ± 0.97
70–74 (72.33 ± 1.20)	21	0.24 ± 0.13	54.90 ± 3.70	28.71 ± 16.50	41.40 ± 9.04	28.90 ± 1.45
75–79 (76.72 ± 1.36)	18	0.25 ± 0.14	54.29 ± 3.42	25.00 ± 5.21	41.18 ± 6.39	*28.06 ± 1.75*
≥ 80 (82.80 ± 2.74)	10	0.21 ± 0.13	49.20 ± 4.02	23.00 ± 5.77	41.90 ± 9.68	28.20 ± 1.23

$\bar{x}$ : mean; SD: standard deviation; FD: framewise displacement; WAIS-Voc: WAIS Vocabulary. The bold indication is $\bar{x}$ and SD represent the mean value and standard deviation of the different measures. BNT, Boston Naming Test; NART, National Adult Reading Test; MMSE, Mini-Mental State Examination.

### 2.6. Estimation of fALFF and ReHo

The estimation of fALFF and ReHo values was performed using DPABI. To estimate ALFF, additional spatial smoothing of the voxels was performed with a 4 mm full width at half maximum Gaussian kernel. After that, the time series of each voxel was transformed to the frequency domain with a fast Fourier transform to compute the power spectrum. To compute ALFF, this power spectrum, with an initial frequency range of 0–0.25 Hz, was square-rooted at each frequency and then averaged across 0.01–0.08 Hz at each voxel. Finally, to obtain fALFF, the latter ALFF values were divided by the whole frequency range observed in the signal (0–0.25 Hz, Zou et al., 2008).

Regarding the ReHo estimation, KCC of the time series of all voxels and their neighbors (n = 27) was calculated (Zang et al., 2004). All ReHo maps were smoothed with a Gaussian Kernel of 4 mm full width at half maximum. Finally, individual fALFF and ReHo maps were standardized into z score maps by subtracting the mean and dividing by the standard deviation.

### 2.7. Statistical analysis

To assess differences within the neuropsychological measures between the six age groups, IBM SPSS (v26) was used to perform ANOVA tests with Tukey’s multiple comparison correction, and *p* < 0.05 was set as significant.

For statistical analysis of the six groups in fALFF and ReHo, DPABI was used with a voxel-wise ANOVA test with Tukey’s multiple comparison correction. As a precautionary measure and to avoid confusion effects head motion, Jenkinson’s FD (Jenkinson et al., 2002), time echo and total gray matter volume were included as covariates in all analyses In addition, the criteria used to assess multiple comparisons was the Gaussian random field (Eklund et al., 2016), with a voxel *p* value of.001 and a cluster threshold of *p* = 0.05. Additional thresholding, *n* = 30 voxels for ReHo and *n* = 10 voxels for fALFF, was set to exclude very small clusters, although they appeared to be significant after the strict Gaussian random field correction.

Moreover, the significant clusters found in the ANOVA test in fALFF and ReHo were extracted using DPABI and were correlated with age and gray matter volume. As the correlations were high a step-wise regression model was adjusted including as predictor variables the gray matter volume and the most common clusters through age groups found in the ANOVA analysis. Moreover, R Studio (R 4.1.2) was used for the correlations, regression analysis, and visualization matrices. In addition, complementary analyses considering age as a quantitative variable were performed to avoid a possible loss of information since age was previously considered a qualitative variable. Therefore, both whole-brain fALFF and ReHo values were correlated with age using the criteria of multiple comparisons with the threshold-free cluster enhancement (TFCE), which reaches the best balance between familywise error and test–retest reliability (Winkler et al., 2016; Chen et al., 2018). A total of 10,000 permutations were performed, and the cluster *p* value was set to *p* < 0.05. As in the previous analyses, there was an additional threshold with a minimum extent threshold of 30 voxels for ReHo and 10 voxels for fALFF.

For the correlation and regression analysis, only the areas which were common for the six groups were chosen in ReHo analysis. Regarding the right caudate and thalamus, as there were both regions regarding these clusters, they were both included in the correlation analysis.

## 3. Results

### 3.1. Participant characteristics analysis

In Table 1, the participants’ movement and neuropsychological measures between age groups are shown.

All of the individuals in our sample had scores higher than 24 on the Mini-Mental State Examination. In addition, no significant differences were observed between the groups as determined by one-way ANOVA in either the National Adult Reading Test [*F*(5, 106) = 0.925, *p* = 0.468], the WAIS-Voc [*F*(5, 99) = 1.095, *p* = 0.368] or the MMSE [*F*(5, 106) = 0.887, *p* = 0.492] or head movement estimated by Jenkinson’s FD [*F*(5,106) = 0.196, *p* = 0.964]. Significantly lower scores in the BNT were detected in the oldest group compared with all of the others [*F*(5, 105) = 3.089, *p* = 0.012, Tukey’s HSD adjusted *p* < 0.001] (Table 1).

Regarding level of education, Table 2 shows the participants characteristics. Group 4 presents 43% of participants with primary studies whereas group 5 presents a 45% of participants with university studies.

**Table 2:** Description of level of education between age groups.

Age groups (years)	Primary (%)	Secondary (%)	University (%)
<60	42	33	25
60–64	38	19	43
65–69	12	44	44
70–74	43	48	9
75–79	33	22	45
≥80	40	20	40

### 3.2. Fractional amplitude of low-frequency fluctuation results between groups

**Figure 1:**
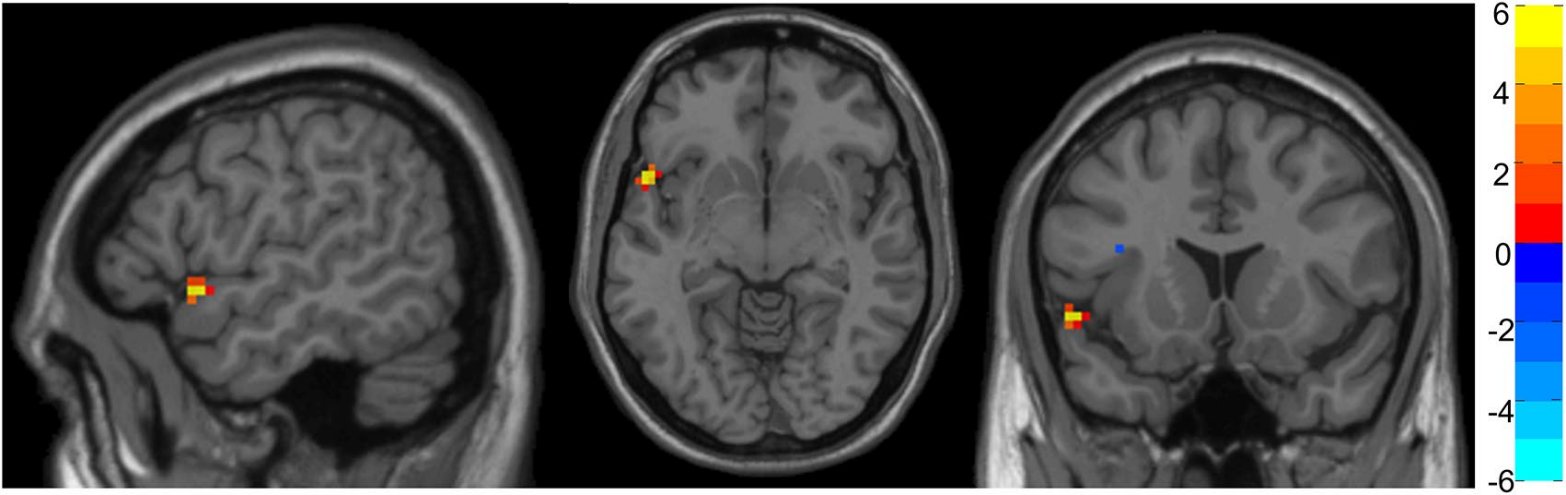
Representation of significant differences between Group 1 and Group 5 in fALFF analysis. The color bar indicates the intensity of the difference, with 6 (yellow) representing positive differences and − 6 (light blue) representing negative differences.

Table 3 shows the significant differences between age groups in fALFF localized within the coordinates of the MNI localized in MNI coordinates and the corresponding brain region defined by the Automatic Anatomical Labeling Atlas (AAL; Tzourio-Mazoyer et al., 2002). Figure 1 shows the graphical representation of the results in fALFF visualized with DPARSF (Yan and Zang, 2010; see footnote 2).

**Table 3:** Significant between-group differences in fractional amplitude of low-frequency fluctuation (fALFF) with their peak localization in MNI coordinates and the corresponding AAL ROI.

Contrast	Area	Number of voxels	*t*(peak)	Peak MNI coordinates (mm)			AAL peak region
Group 1 > Group 5	Superior temporal gyrus and Inferior frontal gyrus	13	4.69	–54	15	–6	Frontal_Inf_Opper_L

MNI: Montreal Neurological Institute; AAL: automatic anatomical labeling.

No significant differences were found between Groups 2, 3, 4, and 6. Nevertheless, Group 1 showed increased fALFF in a cluster of voxels comprehending the superior temporal gyrus and inferior frontal gyrus compared with Group 5.

### 3.3. Regional homogeneity results between groups

**Figure 2:**
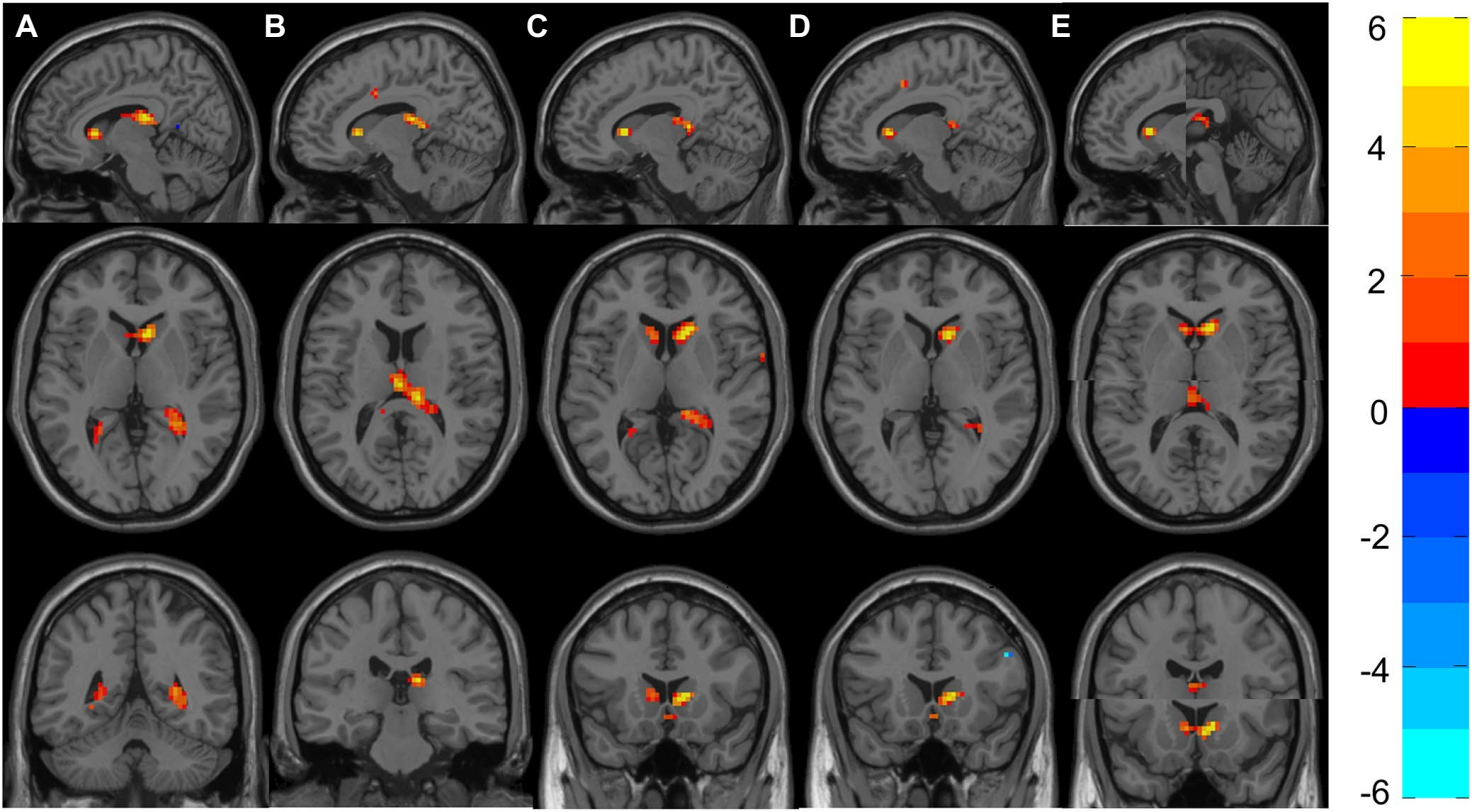
Representation of significant differences between Group 6 and Groups 1–5 in the ReHo analyses. **(A)** Shows the sagittal, axial and coronal planes with the differences between Group 6 and Group 1. Likewise, **(B–E)** Represent the differences between Group 6 against Groups 2–5. **(E)** The two clusters (Right Caudate and Left Thalamus) that appeared to be significant; therefore, two images are shown in each plane. The color bar indicates the intensity of the difference, with 6 (yellow) representing positive differences and –6 (light blue) negative differences.

Table 4 shows the significant differences between groups in ReHo localized in MNI coordinates and the corresponding brain region defined by AAL. Figure 2 shows the graphical representation of the ReHo results visualized with DPARSF (Yan and Zang, 2010; see footnote 2).

**Table 4:** Significant between-group differences in ReHo with their peak localization in MNI coordinates and the corresponding AAL region of interest (ROI).

Contrast	Area	Number of voxels	*t* (peak)	Peak MNI coordinates (mm)			AAL peak region
Group 6 > Group 1	Temporal lobe (hippocampus)	318	5.42	9	−24	15	Thalamus_R
	Right caudate	44	5.31	9	15	3	Caudate_R
	Left precuneus	30	3.96	−24	−48	6	Precuneus_L
Group 6 > Group 2	Temporal lobe (hippocampus)	151	4.98	12	−27	15	Thalamus_R
Group 6 > Group 3	Right caudate	80	5.39	15	18	6	Caudate_R
	Right thalamus and right hippocampus	116	5.11	12	−36	9	Hippocampus_R
Group 6 > Group 4	Right caudate	39	4.94	12	18	3	Caudate_R
	Right precuneus	34	3.87	18	−42	9	Precuneus_R
Group 6 > Group 5	Right caudate	53	5.32	12	15	3	Caudate_R
	Left thalamus	30	4.15	0	−18	15	Thalamus_L

MNI: Montreal Neurological Institute; AAL: automatic anatomical labeling.

All groups showed significant differences with Group 6, where increased ReHo was always found in Group 6 compared with the other groups. More concretely, Group 6 showed increased ReHo in the temporal lobe (hippocampus) compared with Group 1, and they also showed increased activity in the right hippocampus when compared with Group 2. Increased ReHo activation in two separate clusters was found when comparing Group 6 to Group 3: the first showed an increase involving the right caudate, and the other showed an increase in the right thalamus and right hippocampus. Interestingly, Group 6 again showed increased ReHo in the right caudate when compared with Groups 4 and 5.

Moreover, complementary correlation analyses were performed using age as a quantitative variable and whole-brain fALFF and ReHo values. No significant correlations were found between whole-brain fALFF and ReHo values and age.

### 3.4. Correlations and regressions

**Figure 3:**
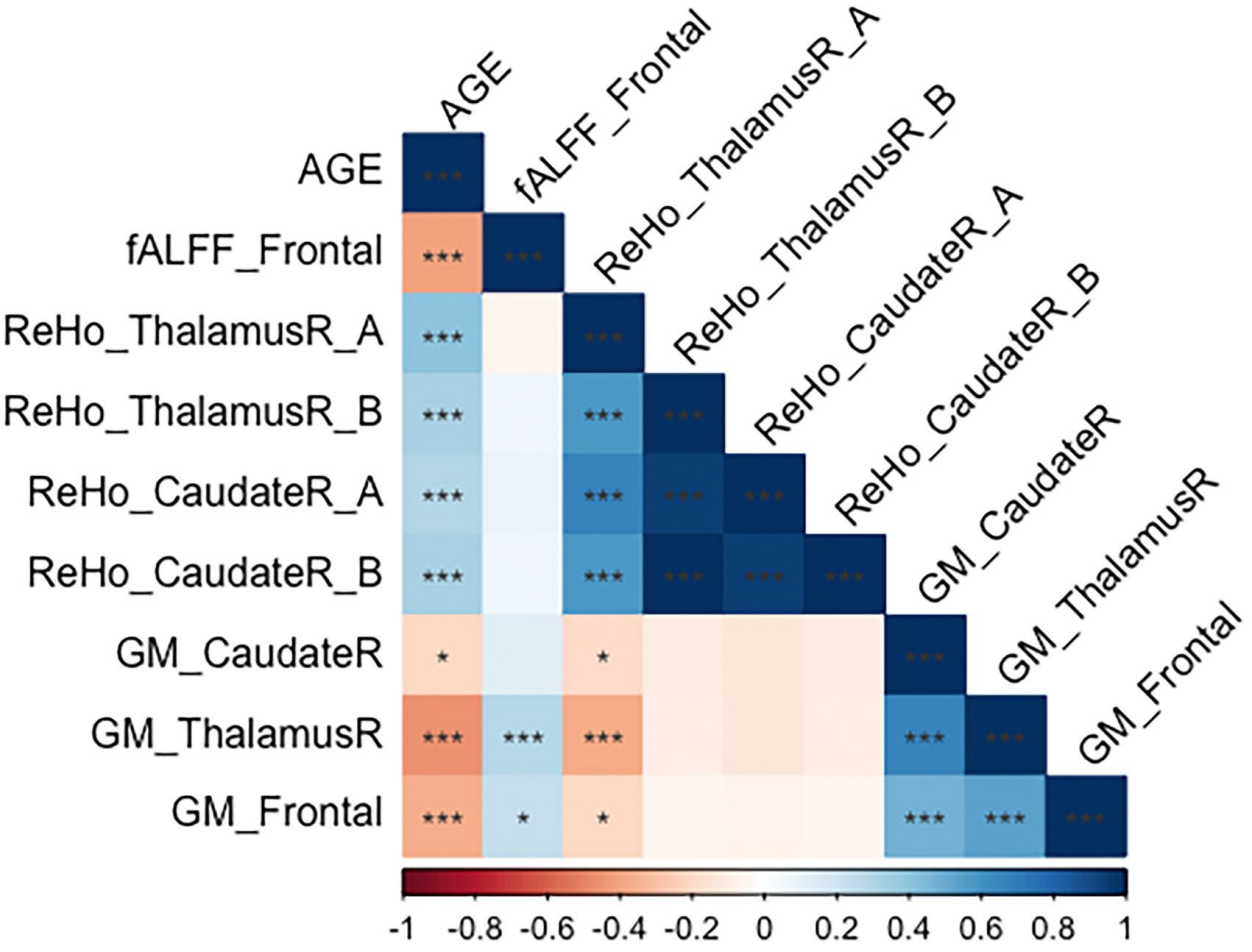
Correlation matrix regarding age, gray matter volume and significant clusters in fALFF and ReHo. ^*^p < 0.05; ^**^p < 0.01; ^***^p < 0.00238 (corrected by Bonferroni).

**Figure 4:**
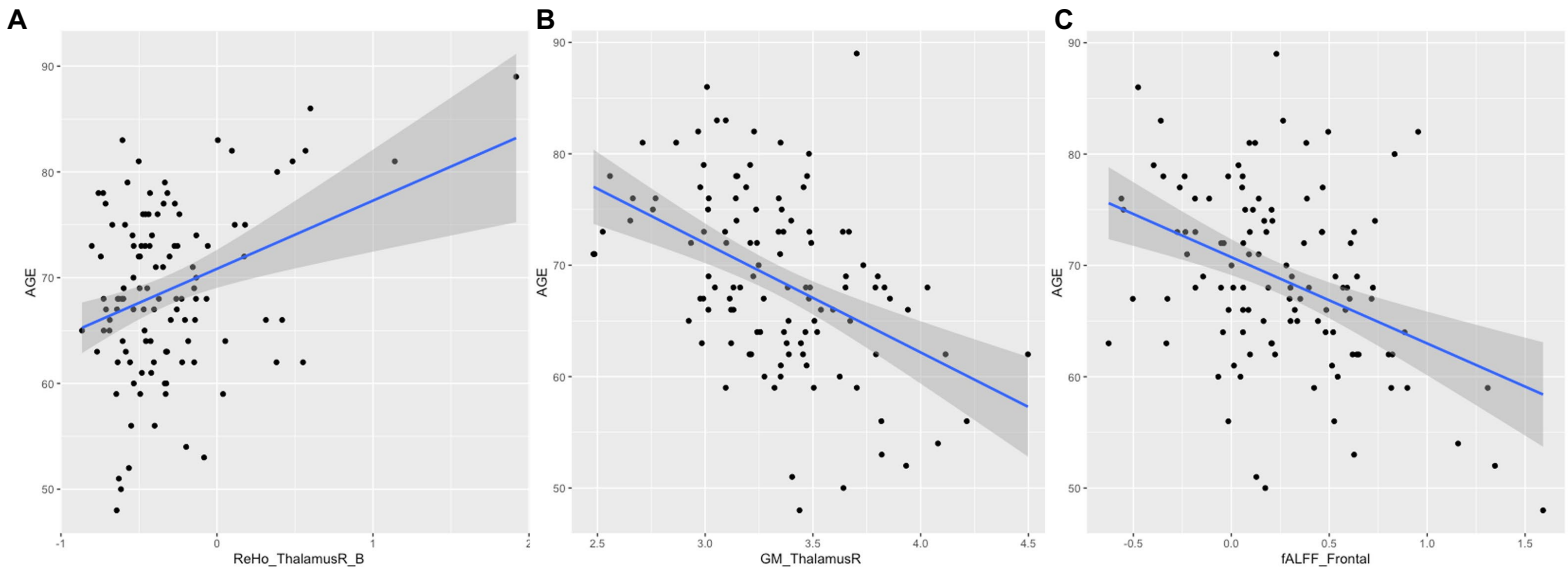
Scatter plot and regression for age. **(A)** Represents age and ReHo signal of the right thalamus; **(B)** Represents age and gray matter volume of the right thalamus. **(C)** Represents age and fALFF signal of the frontal lobe.

Figure 3 shows the correlations between the significant clusters of fALFF and ReHo and the gray matter volume of these specific regions and age. All of them are significant, but it is important to highlight those surviving to the Bonferroni correction. Interestingly, high correlations were found between age and the ReHo signal of both right thalamus clusters, ReHo signal of both right caudate clusters, and their corresponding gray matter volume. Moreover, age is also highly correlated with the fALFF signal of the frontal cluster, as well as their corresponding GM volume. All of them survive to Bonferroni correction, except the GM of the right caudate. In Table 5, the regression model predicting age is presented. The multiple regression meets the conditions (no error autocorrelation, linearity, normality, and homoscedasticity of errors tested). It had high *R*^2^ values, meaning that a high level of prediction was achieved. Only some variables were included as predictors, including fALFF signal of the frontal lobe, ReHo signal of the right thalamus and the right thalamus gray matter volume (Figure 4).

**Table 5:** Parameter estimation (*β*) of the best stepwise linear model for age.

fALFF, ReHo and gray matter	Age
	*F* = 22.52 *R*^2^ = 0.39 AIC = 738.21
Intercept	95.86
fALFF_Frontal	−6.33 (*p* < 0.001)
ReHo Thalamus Right	6.26 (*p* < 0.001)
Gray matter Thalamus right	−7.07 (*p* < 0.001)

They all have a *p* < 0.001 in the model (*df* = 3;108) and a *p* > 0.05 in Anderson’s Darling test of normality, the Ramsey Regression Equation Specification Error (RESET) test, Durbin Watson’s test, and the Breusch–Pagan test.

## 4. Discussion

In the last few years, the importance of healthy aging has increased substantially due to the higher levels of life expectancy in the population and due also to the appearance of dementia in the general population, which dramatically increases societal costs. Therefore, disentangling the brain mechanisms of healthy aging has been a subject of interest for researchers. The aims of the present study were to study the whole-brain resting state using fALFF and ReHo strategies to explore differences in spontaneous brain activity among healthy participants of different age groups from middle to advanced age.

Regarding the whole-brain fALFF analysis, the results showed significant differences in low-frequency fluctuations between Groups 1 and 6 in the superior temporal gyrus and inferior frontal gyrus (Table 3). More concretely, group one (the youngest one) shows increased fALFF in this area.

These results are in line with those reported by other authors. The superior temporal gyrus appeared to show a volume decrease (atrophy) in healthy aging, revealing a relationship between age and the rate of atrophy (Fjell et al., 2009). This atrophy could also explain the decrease in fALFF values among this population. Moreover, Galiano et al. (2020) also found higher intrinsic connectivity contrast and higher cerebral blood flow between young and elderly groups. Oschmann et al. (2020) also reported significantly decreased FC in this area in a longitudinal study (4-year follow-up) when using the right inferior parietal sulcus as a seed. The inferior frontal gyrus, which is an area involved in language functions, has also been found to have decreased gray matter density in older adults (Pistono et al., 2021).

Moreover, the detected cluster appears to be disrupted by aging, and the significant differences would be expected to involve the oldest group. However, the differences found in this study involve the second oldest group (Group 5) and not the oldest group. This fact is consistent with Farras-Permanyer et al. (2019), who suggested that this phenomenon could be a survival mechanism, meaning that the participants of Group 6 would have a high degree of resilience.

In relation to the ReHo results, significant differences between all groups and Group 6 were found, indicating higher synchronization of rs-fMRI signals among neighboring voxels in Group 6. More specifically, Group 6 had increased ReHo values in some areas of the temporal lobe (hippocampus) compared with Groups 1, 2. However, this cluster has different volumes depending on the groups compared. It has its maximum size (318 voxels) when comparing age Groups 1 and 6, and as the age of the participants increases, it decreases to 151 voxels when comparing Groups 2 and 6. It is important to highlight that, even if it is smaller, the cluster is significant also in group 3 (involving the right thalamus and right hippocampus, 116 voxels). As individual effects, it is also important to remark that group 6 shows increased ReHo compared with group 1 in the left precuneus whereas group six shows increased ReHo in the right precuneus. The left thalamus is increased in ReHo values when comparing group 5 and 6. All these structures which show significant group differences among age groups include the DMN, which is one of the intrinsic resting-state networks that has been most studied with respect to aging.

Fjell et al. (2014) highlighted the hippocampus as a vulnerable region to aging. The FC of the hippocampus also decreases in Alzheimer’s disease (AD; Greicius, 2008; Sheline et al., 2010). In healthy elderly individuals, Bartrés-Faz et al. (2008) examined the FC of the hippocampus during an encoding memory task and found increased connectivity with the anterior cingulate, inferior parietal lobe, and caudate in APOE-ε4 carriers. Oschmann et al. (2020) points out the importance of other networks, as the frontoparietal network, apart from the DMN. Despite these unclear conclusions (Hu et al., 2014), the hippocampus appears to be a key structure that changes with age. It is clear that a broad individual heterogeneity emerges in this population; Abellaneda-Pérez et al. (2022) proposes a combination of noninvasive brain stimulations (NIBS) and fMRI to understand how fundamental brain plasticity mechanisms operate in advancing age.

The second cluster (right caudate) shows increased ReHo values in Group 6 compared with Groups 1, 3, 4 and 6. However, as shown in Table 4, the size of this second cluster varies through age group comparisons which is higher on Group 3. These results are also in line with those reported in other studies. Bennett et al. (2011) found age-related decreases in caudate-dorsolateral prefrontal cortex tract integrity that mediated age-related differences in late-stage sequence learning. In a task-fMRI study, Bowen et al. (2020) found that older but not younger adults exhibited enhanced subsequent memory for high-reward items, supported by greater connectivity between the caudate and bilateral inferior frontal gyrus. Tang et al. (2021) found decreased FC within the right caudate and some regions of the cerebellum in AD.

Our results show that even controlling by gray matter volume, fALFF and ReHo show significant differences in healthy aging. The correlation matrix demonstrates the clear relationship between age and neuroimaging signal, beginning with ReHo signal in the right thalamus, which correlates positively with age. The ReHo signal of the right caudate also correlates positively with age. Nonetheless, fALFF signal in the frontal cluster is negatively linked with age, showing a decrease of fALFF in these areas. Remarkably, structural changes are also associated with age, finding a negative correlation with this variable. In this sense, gray matter volumes of the right thalamus, the right caudate and the frontal cluster are negatively associated with age, having therefore decreased volume as age advances. These structural abnormalities have also been reported in Pergher et al. (2019). They also show negative correlations with sociodemographic variables such as age. As high correlations were found between these measures and age, we performed step-wise regression to try to predict age by neuroimaging data. Results show a high variability explained by these structural and spontaneous brain activity measures, suggesting its utility as biomarkers of age. However, more studies are needed to demonstrate its potential. Other authors have suggested already the ability of fALFF and ReHo measures as potential biomarkers owing to their high test–retest reliability (Küblböck et al., 2014; Zuo and Xing, 2014).

Both techniques have been shown to be valuable and usable tools for disentangling brain changes in activation in different groups of healthy aging. fALFF and ReHo techniques measure different outcomes in the brain; therefore, differences in groups estimate two features involved in aging. On the one hand, the fALFF results indicate a significant difference in low-frequency fluctuations between Groups 1 and 5 despite not being translated into a significant change in performance in terms of the neuropsychological measures. However, interestingly, this result is in line with the results of Farras-Permanyer et al. (2019) using the same sample concerning progressive FC decrease either in number or intensity of connections. On the other hand, the ReHo results indicate an increase in regional synchronization between Group 6 and the other age groups. Other studies have also reported increased ReHo measures and have considered it to offset functional decrease or impairment, i.e., a compensatory mechanism (Zhang et al., 2012; Song et al., 2014; Kong et al., 2015). This finding may be crucial because it directly links the increase in ReHo signal with healthy aging; furthermore, there is no evidence of impairment and no decrease in the neuropsychological performance. The differences in the findings of fALFF and ReHo, which remained in Groups 5 and 6, could be linked to the fact that both groups were healthy aging participants without any suspicion of dementia or any other cognitive decline. Meanwhile, Group 6 included more elderly individuals who therefore have higher resilience. These results demonstrate that increased ReHo values could be directly linked with compensatory mechanisms due to brain aging.

This study has some limitations. First, an important limitation is found in the sample size. Although the study cohort contains 112 participants, the size of each age group was not entirely homogeneous, leaving the oldest group with 10 participants. Therefore, sample size, distribution and dispersion may introduce a bias in the final results, especially concerning the older group. The lack of a replication dataset could also have limited the results. Second, low-frequency BOLD signals, especially in the brain regions that comprise the DMN (Birn et al., 2006), are affected by physiological noises (Birn et al., 2008; Chang and Glover, 2009). We cannot truly assess the impact of these physiological noises or any blood pressure-induced hemodynamic response fluctuation as we did not collect respiratory and cardiovascular data. Finally, motion, even if well controlled, might affect the results.

Some strengths of the study are also worth mentioning. As there is a fundamental need to better understand the neurobiological changes associated with healthy aging given the globally aging population, elucidating the differences in spontaneous brain activity between age groups of healthy aging is of major importance. This study demonstrates that changes in spontaneous brain activity may occur in very low intervals of years, and those changes could be targeted as specific therapeutic areas in cognitive rehabilitation. Finally, a highly restrictive correction for multiple comparisons was performed in this analysis to ensure that the strongest differences remained significant. The results have a large effect size, so we can affirm that significant differences in regional spontaneous brain activity using fALFF and ReHo were found between the six groups of an elderly population. Finally, the strong relationship between age and structural and spontaneous brain activity measures suggests the possibility of using them as potential biomarkers.

## Data availability statement

The data analyzed in this study is subject to the following licenses/restrictions: The data are not publicly available due to privacy or ethical restrictions. The data that support the findings of this study are available on request from the corresponding author. Requests to access these datasets should be directed to mmontala@ub.edu.

## Ethics statement

The studies involving human participants were reviewed and approved by Comisión de Bioética de la Universitat de Barcelona Servicio de Farmacia del Hospital Clínic de Barcelona Comité Ético de Investigación Clínica (CEIC) Comité Investigación del Hospital Clínic de Barcelona. The patients/participants provided their written informed consent to participate in this study.

## Author contributions

All authors contributed to the study's conception and design. LV-A and DB-F curated the data. DB-F and JG-O provided the resources for the study. MM-F and CC-M made the first conceptualizations of the paper, investigation, and methodology. Supervision of the paper was performed by MP-C and JG-O. All authors contributed to the article and approved the submitted version.

## Funding

This work was supported by the Walnuts and Healthy Aging (WAHA) study (Grant number NCT01634841) funded by the California Walnut Commission, Sacramento, California, United States and the Spanish Ministry of Science, Innovation and Universities, Agencia Estatal de Investigación (Grant Number: PGC2018-095829-B-I00 and MICIU/FEDER; RTI2018-095181-B-C21). DB-F was supported by an ICREA Academia 2019 award.

## Conflict of interest

The authors declare that the research was conducted in the absence of any commercial or financial relationships that could be construed as a potential conflict of interest.

## Publisher’s note

All claims expressed in this article are solely those of the authors and do not necessarily represent those of their affiliated organizations, or those of the publisher, the editors and the reviewers. Any product that may be evaluated in this article, or claim that may be made by its manufacturer, is not guaranteed or endorsed by the publisher.

## Footnotes

^1^ http://www.fil.ion.ucl.ac.uk/spm

^2^ http://rfmri.org/DPARSF

## Abbreviations

AD: Alzheimer’s disease
BNT: Boston naming test
BOLD: blood-oxygen-level-dependent
DMN: default mode network
fALFF: fractional Amplitude of Low-Frequency Fluctuations
FC: functional connectivity
FD: framewise displacement
MMSE: mini-mental state examination
MNI: Montreal Neurological Institute
NART: national adult reading test
RAVLT: Rey auditory verbal learning test
ReHo: regional homogeneity
ROI: regions of interest
Rs-fmri: resting state functional Magnetic Resonance Imaging
VBM: voxel based morphometry
WAIS: Weschsler adult intelligence scale
